# Erratum for “Long‐Term Outcomes of Mitral Valve Repair With Artificial Chordae and Annuloplasty for Myxomatous Mitral Valve Disease in Dogs”

**DOI:** 10.1111/jvim.70186

**Published:** 2025-07-24

K. Kurogochi, S. Furusato, E. Takahashi, M. Tabata, M. Mizuno, Y. Nii, and M. Uechi. “Long‐Term Outcomes of Mitral Valve Repair With Artificial Chordae and Annuloplasty for Myxomatous Mitral Valve Disease in Dogs,” *Journal of Veterinary Internal Medicine* 39, no. 4 (2025), https://doi.org/10.1111/jvim.70171.

In the above‐mentioned article, Figure 1 “Time‐to‐event curves following mitral valve repair compared among clinical stages” in panels (A) and (B), the ACVIM Stage labels should display “D” in third row not “2.” The correct figure is displayed below.

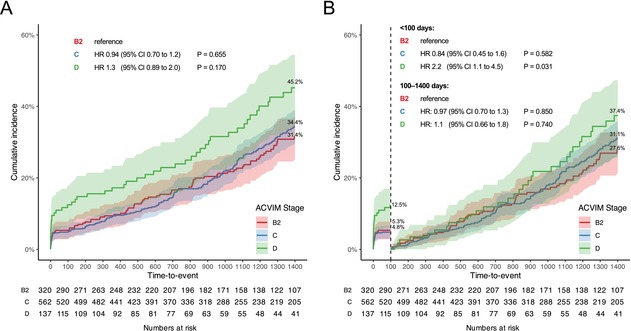

We apologize for this error.

